# Sperm Antioxidant Biomarkers and Their Correlation with Clinical Condition and Lifestyle with Regard to Male Reproductive Potential

**DOI:** 10.3390/jcm9061785

**Published:** 2020-06-08

**Authors:** Wirginia Krzyściak, Monika Papież, Ewelina Bąk, Eva Morava, Paweł Krzyściak, Anna Ligęzka, Agnieszka Gniadek, Palina Vyhouskaya, Jarosław Janeczko

**Affiliations:** 1 Department of Medical Diagnostics, Faculty of Pharmacy, Jagiellonian University Medical College, Medyczna 9, 30-688 Krakow, Poland; ligezka.anna@mayo.edu (A.L.); palina.vyhouskaya@gmail.com (P.V.); 2 Department of Cytobiology, Faculty of Pharmacy, Jagiellonian University Medical College, Medyczna 9, 30-688 Krakow, Poland; monika.papiez@uj.edu.pl; 3 PARENS Infertility Clinic, 29 Listopada 155C, 31-406 Krakow, Poland; ewelina.bak@vp.pl (E.B.); biuro@parens.pl (J.J.); 4 Department of Clinical Genomics, Center for Individualized Medicine, Mayo Clinic, Rochester, MN 55905, USA; morava-kozicz.eva@mayo.edu; 5 Department of Mycology, Jagiellonian University Medical College, Czysta 18, 31-121 Krakow, Poland; pawel.krzysciak@uj.edu.pl; 6 Department of Nursing Management and Epidemiology Nursing, Faculty of Health Sciences, Jagiellonian University Medical College, Kopernika 25, 31-501 Krakow, Poland; agnieszka.gniadek@uj.edu.pl

**Keywords:** male infertility, oxidative stress, antioxidant potential, reduced glutathione

## Abstract

Measurement of sperm oxidative-antioxidant indicators is widely used in the assessment and detection of biochemical causes of male infertility. The main purpose of this study was to identify biomarkers that assist in diagnostics and monitoring of male reproductive potential. We performed the assessment of oxidative-antioxidant malondialdehyde (MDA), glutathione (GSH), and total redox antioxidant potential (TRAP) indicators in seminal plasma, seminogram, clinical condition, and lifestyle of people with reproductive problems. The combined assessment of GSH and TRAP as potential biomarkers of male infertility in semen plasma was characterized by the highest total sensitivity and specificity. Furthermore, we provide evidence that male reproductive potential is significantly correlated with basic sperm parameters, sperm cell membrane integrity, their morphology, lifestyle, eating habits, occupation, and mental health. Our results provide evidence on the importance of oxidative stress and defense against free radicals in diagnosing and monitoring men with infertility that are consistent with previously conducted research. We provide an alternative approach on the possibility of interpreting the combination of the biomarkers that can bring benefits to a multi-threaded approach to the diagnosis and treatment of male infertility.

## 1. Introduction

The World Health Organization’s (WHO) defines infertility as the inability to conceive despite regular unprotected intercourse during 12 months or longer [1]. A couple’s fertility is diagnosed based on various reasons, including a man’s age being more than 35 years old and a woman’s age above 30 years old, with diagnosis of conditions, such as oligomenorrhea or amenorrhea, and with pathological changes in the reproductive organs, as well as men exhibiting infertility attributed to female factors (approximately 1/3 of infertility) [2]. Currently, nearly 48.5 million couples have a problem with infertility worldwide, and almost 15% of them have been trying to conceive a child [3]. A total inability of a man to fertilize a woman occurs at a frequency of 2% and is defined as a primary infertility [4,5]. Secondary infertility affects 11% of the population and is defined as an inability to re-fertilize a partner who has had a pregnancy at least once, or if the pregnancy ended in a miscarriage [6]. In Poland, nearly 40% of 1.5 million registered couples struggling with infertility try to solve this problem using primary health care, while 60% require specialist treatment as assisted reproduction techniques (ART), performed in 2% of infertile couples [7,8,9].

Furthermore, the average age of an infertile male is about 33 years old, where the duration of infertility reaches 3 years [10]. The share of the male factor in infertility is close to that of women (ca. 50%), while about 20% of infertility cases involve overlapping male and female factors. Idiopathic infertility remains to be a major problem in all cases of not-recognized male infertility [5,11]. 

Technological progress that has taken place in the last decade; thus, the emergence of more diagnostic tools that can highlight problems with sperm has enabled a more complete assessment of male reproductive functions. 

Widely appearing literature data combining the topic of oxidative stress (OxS) with the problem of infertility constitute an evidence of a never-ending interest in the subject [12,13,14]. Excess of reactive oxygen species (ROS) damages mitochondrial and nuclear DNA, causes lipid peroxidation, reduces sperm motility, increases the risk of male infertility, and even may be a reason of congenital malformations in the progeny [15,16]. OxS is characterized by an imbalance between increased sperm injury caused by reactive oxygen species (ROS) and the inability of antioxidative agents to remove these [3,17]. Despite the adverse role of oxidative stress reducing reproductive function in men, ROS also play a physiological role in sperm maturation [18], i.e., participate in the initiation of their basic life functions, such as the ability to penetrate and fertilize, acrosomal response, hyperactivation, chemotaxis, and motility [19,20]. 

WHO recommends a seminogram in male fertility evaluation, which is a general examination of semen; however, this does not always enable the determination of the cause of infertility and spontaneous abortions and pregnancy losses after in vitro procedures, inter alia, after intracytoplasmic sperm injection (ICSI). The quality of sperm and oocytes plays a decisive role in the ICSI fertilization, in which OxS may participate. 

It has been demonstrated that a 25% increase in ROS production in seminal fluid activates cellular response, OxS induction, and apoptosis in the p53-dependent pathway associated with inhibition of natural antioxidants, such as GSH, superoxide dismutase (SOD), catalase (CAT), or glutathione peroxidase (GPx). Increased OxS, co-accompanying other overlapping factors, reduces the ability of sperm to reproduce [12,16,21,22]. Damage to mitochondrial DNA impairs the maintenance of the fertilized ovum and permanently disturbs the genetic material passed on to the progeny. This is important in maintaining fertilized embryos and reducing the still high number of abortions after in vitro procedures [23,24].

The main goal of this study was to assess the usefulness of selected indices of OxS and antioxidant defense in seminal plasma as biomarkers of semen quality in male infertility. The assessment of their correlation with such semen parameters as mobility, concentration, morphology, and vitality, as well as questionnaire data, may contribute to improving the knowledge on etiopathogenesis of male infertility and the effectiveness of its treatment. That can allow better identification of men at risk of infertility without any visible clinical signs, prediction of semen damage caused by ROS, and its prevention, resulting in pregnancy and the birth of a healthy child in patients with infertility. 

Although the parameters of oxidative stress tested so far have not been supported by sufficient scientific evidence as biomarkers of male infertility, they are still among its pathogenetic factors available in the recommendations of international urological, gynecological, or reproductive medical associations [25,26,27,28], which allows concluding that their significance is unquestioned.

The obstacle in recognizing the parameters of oxidative stress as biomarkers of male infertility is the lack of automated methods for measuring free radical damage, short half-life (T_1/2_) of free radicals, as well as lack of a combined assessment of individual oxidant-antioxidant parameters with clinical factors, and environmental conditions. Therefore, it can be expected that only a comprehensive set of oxidative stress parameters and other recognized predictors of male infertility, such as seminogram assessment [29], sperm DNA fragmentation (SDF) [30], contribution of environmental factors (smoking, extremely high and low temperatures, and viral infections) [31,32,33], and eating habits, can provide information on dynamic changes in male gonad activities and reproductive potential during and after the occurrence of these factors. 

In many cases, laboratory test performance is not particularly thought out but is a matter of habit and is required for documentation purposes. Making decisions about the use of predictors to help assess sperm quality in couples seeking offspring is generally difficult. The situation is even more complicated when it is necessary to make a decision in conditions of uncertainty. That is why we tried to enrich the literature on the subject of oxidative stress and male infertility by the cut-off points for the limit values of the studied parameters (total redox antioxidant potential (TRAP), glutathione (GSH), or malondialdehyde (MDA)), which, in our opinion, can be a starting point in determining the hierarchy of importance for potential causes of male infertility. In addition to these biomarkers, we draw attention to a multidisciplinary approach using their reference to medical history and a questionnaire interview, together with an assessment of the impact of seminogram parameters and environmental factors, which, when considered together, can provide information on the course and effects of male infertility in the future.

To the best of our knowledge, there is no literature data presenting a combined approach on the importance of several biomarkers together (TRAP, GSH, and MDA) among men with infertility problems nor in the group of couples planning in vitro fertilization. 

Therefore, the aim of the study was to assess the diagnostic usefulness of the combined approach of several potential predictors of male infertility. For the first time, we combine several oxidative stress parameters with the markers other than seminogram, indicating further research directions related to the monitoring of male reproductive potential (through the combined assessment of oxidant-antioxidant indicators and determination of their relationship with the parameters already used in clinical decision-making regarding male infertility).

## 2. Experimental Section

The subjects enrolled in this study are volunteer participants and were recruited from Parens Infertility Treatment Center in Krakow from December 2017 to May 2019. Written informed consent were obtained by gynecologists and andrologists with relevant clinical experience from all study participants and their partners. Each of them was informed about the purpose of this study. The study was approved by the Bioethical Committee of the Jagiellonian University in Krakow (approval no: 1072.6120.265.2017 of 21 December 2017, including the extension of the study until May, 2019). The clinical examination was conducted in accordance with the principles of the Helsinki Declaration of 2008. Infertile men were recruited into the study group who reported to the Infertility Clinic due to repeated unsuccessful attempts to conceive a child for at least 12 months or due to repeated unsuccessful intrauterine inseminations without any apparent reason. Males presenting to the Infertility Clinic were screened and enrolled in the study if they experienced repeated unsuccessful attempts to conceive a child for at least 12 months or due to repeated unsuccessful intrauterine inseminations without any apparent reason.

The exclusion criteria include the factors clearly affecting the oxidative-antioxidant status, i.e., genetic diseases, microbial infections, stimulants (alcohol, drugs, marijuana); age < 20 or > 40; use of the following drugs in the last three months: antibiotics, nonsteroidal anti-inflammatory drugs, corticosteroids, vitamins, dietary supplements; inflammation of the genitals or abdominal cavity; diseases and inflammations of the genitourinary tract in the last 6 months; surgery; metabolic syndrome features (insulin resistance, hypertension, atherosclerosis, or hyperlipidemia); or withdrawal of a patient from the study without providing a clear reason. Men whose clinical history showed an increased susceptibility to infertility due to the use of hormonal pharmacotherapy or exposed to occupational factors generating mental or oxidative stress were also excluded from the study.

The control group comprised men who had offspring (one or two children) a year or two before the examination and reported to the Infertility Clinic to perform control tests at the same time. They were followed up in connection with an earlier history of infertility and were included in the study due to the current lack of negative changes in sperm parameters and success in conceiving children. Data were prospectively collected from participants during the study, and questionnaire data was collected (Supplementary Material S1), which included questions about age, sexual history, lifestyle, diet, psychosocial conditions, past diseases including autoimmune diseases, metabolic syndrome, and genetic diseases.

Twelve men with oligozoospermia, 11 with teratozoospermia, and 15 with asthenozoospermia were recruited to the study group. The qualification was based on the international guidelines of the World Health Organization on semen analysis [2], which define oligoozoospermia as the total number of spermatozoa < 39 million per ejaculate; teratozoospermia as the share of spermatozoa with normal structure < 4%; and asthenozoospermia as progressive motility < 32% (regards spermatozoa that do not swim quickly with a progressive movement, which reduces the likelihood of distant fertilization of a female ovum in the genital tract).

In total, 113 individuals were recruited and divided into two groups: patients with infertility and controls with offspring. Demographic data, i.e., the average age of fertile and infertile men, their partners’ age, height, weight and, body mass index (BMI) were assessed in the groups (Table 1).

Sperm samples were obtained by masturbation 2–5 days after the last ejaculation, after a night’s rest. Each semen sample was collected according to the 2010 WHO guidelines on sperm testing [2].

In addition to manual assessment of the seminogram, the material was analyzed using computer sperm analysis (CASA). Seminogram and cell membrane integrity were evaluated in fresh ejaculate, while oxidative and antioxidative parameters in the seminal plasma were evaluated after obtaining statistically insignificant differences between these parameters between the seminal plasma and the entire ejaculate.

A sperm cell membrane integrity analysis was performed on a flow cytometer using the LIVE/DEAD Sperm Viability Kit (Thermo Fisher, Munich, Germany) according to the manufacturer’s instructions within 0.5 h of material collection. 

The liquefied semen was centrifuged at 300× *g* for 10 min at a room temperature. The remaining sperm in a volume of approximately 1 mL was washed twice in 3 mL of sperm washing medium (HEPES—buffered human tubal fluid (HTF) medium supplemented with human serum albumin, Irvine Scientific, Santa Ana, CA, USA). Afterward, 0.5 mL of medium was added to the suspension, and the falcon tilted at a 45° angle was incubated at 37 °C for at least 45 min. The falcon was then placed in a vertical position and used for multiparameter analysis on a flow cytometer.

### 2.1. Lipid Peroxidation Product; MDA—Malonyldialdehyde

The concentration of lipid peroxidation products in the seminal plasma was assessed based on the method developed by Buege and Aust [34] and modified by Gutteridge [35]. The method is used to determine one of the main lipid peroxidation products—malonyldialdehyde (MDA) reacting with thiobarbituric acid (TBA). Subsequently, the resulting MDA-TBA adduct was extracted with n-butanol and the fluorescence in the organic layer was measured, which further increased the specificity of the method [36].

Semen samples (containing approximately 5 × 10^6^ spermatozoa) were prepared by suspending them in tris-citric acid buffer (TCF, 0.05 mM, pH 7.4) to a final volume of 0.4 mL.

MDA concentration was determined by comparing the fluorescence intensity of the sample at absorption and emission wavelengths with a standard calibration curve using the malonyldialdehyde equivalent formed in the presence of 1,1,3,3-tetramethoxypropane (TMP). The results are expressed in µmol MDA/mL seminal plasma.

### 2.2. Total Redox Antioxidant Potential (TRAP) 

TRAP was determined in the semen plasma using the Benzie and Strain method based on the ability of Fe^3+^ to Fe^2+^ reduction in the presence of the tripyridyltriazine (TPTZ) [37]. 

### 2.3. Reduced Glutathione (GSH)

The total concentration of thiol groups (GSH) in the seminal plasma was determined according to the method of Riddles et al. [38]. Most thiol groups were determined with 5,5’-dithio-bis-(2-nitrobenzoic acid) (DTNB), resulting in the formation of a yellow 5-thio-2-nitrobenzoic acid (TNB) dianion, which was the subject of a measurement [39,40].

GSH in the samples was calculated by comparison with a standard calibration curve consisting of known cysteine concentrations.

### 2.4. Total Protein Levels

Total protein (TP) concentrations of seminal plasma samples were measured by the bicinchonic acid (BCA) method according to the instructions of a manufacturer (Sigma-Aldrich, Saint Louis, MO, USA) (bovine serum albumin (BSA) constituted a standard). 

The integrity of the spermatic membrane was assessed on a flow cytometer with a LIVE/DEAD Sperm Viability Kit according to the instructions of a manufacture, using live sperm cells fluoresced green and propidium iodide red (Thermo Fisher). The live sperm cells fluoresced green (SYBR 14) fluorophore passes through the intact plasma membrane of living cells and binds to DNA, and propidium iodide (PI) stains the DNA of cells with a damaged cell membrane red. The beginning of dying process can be recognized after the simultaneous staining of cells with two fluorophores. SYBR 14 was prepared freshly by making a 5 μL dilution of the basic solution in 120 μL of sterilized and filtered HBSS (Hank’s balanced salt solution); PI was used undiluted. Pre-diluted sperm samples were diluted again (1:200) in HBSS and each stain was added at a final concentration of 6.7 nM SYBR 14 and 3.3 M PI. The analysis was carried out after 5 min of incubation in the dark at 37° (21 to 25 ± 1° C), in a flow cytometer (BD LSR II, Becton Dickinson, Biosciences Immunocytometry Systems, San Jose, CA, USA) equipped with band filters: 530/30 nm for SYBR-14 and 575/26 nm for PI. Flow cytometer settings were adjusted using a positive (100% dead cells) and negative (unstained fresh sperm) controls. Ten thousand events were calculated for each sample. 

### 2.5. Statistical Analysis 

A comparison of the values in both groups was executed using the Student’s *T* test (when the variable had a normal distribution in these groups) or the Mann–Whitney test (otherwise). The comparison of the values of quantitative variables in three or more groups was performed using the ANOVA analysis of variance (when the variable had a normal distribution) or the Kruskal-Wallis test (otherwise). After the detection of statistically significant differences, a post-hoc analysis was performed with the Fisher’s least significant difference (LSD) test (in the case of normality of distribution) or Dunn’s test (in the absence of normality), in order to identify statistically different groups. Correlations between the selected variables were calculated using Pearson’s (when both were normal) or Spearman’s (otherwise) correlation coefficient. The strength of the relationship was expressed as follows: |r| ≥ 0.9—very strong relationship, 0.7 ≤ |r| < 0.9—strong relationship, 0.5 ≤ |r| < 0.7—moderately strong relationship, 0.3 ≤ |r| < 0.5—weak relationship, and |r| < 0.3—very weak (negligible) relationship. The cut-off point for tests based on quantitative variables was determined on the basis of the receiver operating characteristic (ROC) curve, choosing the point closest to the upper left corner of the graph. The suitability of the quantitative variable as a predictor was evaluated using the area under the ROC curve (AUC). The normality of the variable distribution was assessed using the Shapiro-Wilk test. The analysis assumed a 0.05 significance level. Thus, all *p* values below 0.05 were interpreted as indicating significant relationships. The assumed level of confidence for selected was established as α = 0.05 and test power was >90%. The analysis was carried out in the R program, version 3.5.0. 

## 3. Results

### 3.1. Demographic Analysis 

The average age of people in fertile and infertile groups was 37.5 ± 2.46 and 34.53 ± 3.95 years, respectively (Table 1). Due to the fact that there were no statistically significant differences between men with oligoozoospermia, teratozoospermia, and asthenozoospermia, further comparisons were carried out in the groups of infertile men and fertile men as controls.

The only significant differences among age, age of a partner, height, weight, and BMI were the age of the patient, the age of their female partners, and their BMI (lower in the group of infertile men) (Table 1).

### 3.2. Questionnaire Data

Analyzing the questionnaire data, i.e., work in variable temperature conditions, TRAP levels were significantly higher in men working at lower temperatures compared to TRAP levels in men working at higher temperatures. The presence of any harmful factor had the same impact on the TRAP parameter level. The type of performed work (sitting or standing) had no significant effect on the measured parameters. 

In the context of physical activity, statistically significant changes in the total sperm count were found in men who practice sports every day compared to those who are active rarely. The frequency of practicing physical activity also had a statistically significant impact on the level of TRAP, MDA, and total protein. TRAP and protein levels were higher in men who regularly exercise than in those who do it sporadically. The comparison of research results in this matter is, however, difficult because the literature on the subject assessing the impact of physical effort on the quality of sperm so far reports inconsistent results [41].

Significant differences were observed between TRAP, GSH, and MDA levels and drinking coffee and the consumption of fruits and vegetables. Frequent consumption of fruits and vegetables and rare coffee drinking resulted in a higher antioxidative potential expressed as TRAP and GSH and lower values of MDA (Supplementary Material S1: Questionnaire data. Study questionnaire—results. Drinking coffee; Fruits and vegetables intake).

Having children also influenced the parameters of the seminogram and the values of oxidative-antioxidant parameters. Thus, amongst couples having offspring but having problems with the conception of another child, MDA was lower than in men from couples having problems with getting pregnant for the first time. The period of applying for a child positively correlated with the level of MDA and negatively correlated with the level of TRAP and GSH (Supplementary Material S1: Questionnaire data. Study questionnaire—results. Having offspring).

### 3.3. Analysis of Basic Semen Parameters 

**Figure 1:**
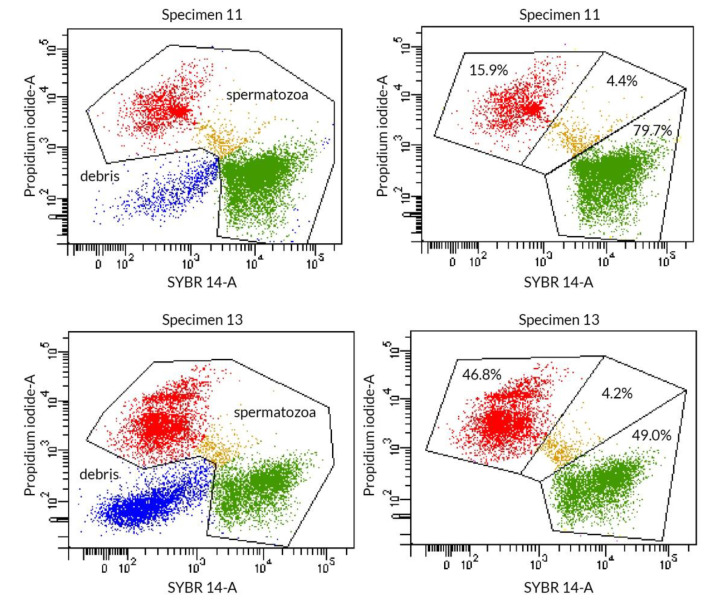
Examples of flow cytometer charts concerning sperm cell integrity in fertile (sample no 11) and infertile men (sample no 13). SYBR 14 and propidium iodide (PI) staining. Red—dead spermatozoa with impaired cell membrane integrity; yellow—double positive spermatozoa beginning to lose the integrity of the cell membrane; green—viable spermatozoa with intact cell integrity; blue—debris or fragments of broken spermatozoa.

The analysis of standard parameters of sperm, e.g., concentration, total sperm count, motility, morphology, and cell membrane integrity and viability of both groups showed that significantly lower values of the following parameters were observed in the infertile group: concentration, motile sperm progressive cells, plasma membrane integrity (Figure 1), viability, and normal morphology, while non-moving motility of sperm was higher than in the fertile group (Table 2). 

Out of 38 patients, the number of those with oligozoospermia, teratozoospermia, and asthenozoospermia was equal to 12, 11, and 15, respectively. No statistically significant differences were found between men with oligoozoospermia, teratozoospermia and asthenozoospermia (confidence interval α = 0.05); hence, further analyses were performed in the groups of infertile (patients) and fertile (controls) men.

### 3.4. Analysis of Selected OxS Indices 

Before the selection of plasma for the study of oxidative stress parameters, pilot studies were performed on a group of 15 infertile and 15 healthy individuals, comparing these parameters in the seminal plasma and the spermatozoa alone. Due to the fact that no statistically significant differences were found in the oxidation-antioxidant parameters studied between the seminal plasma and the spermatozoa themselves, seminal plasma was used for further analysis.

MDA in seminal plasma of infertile patients was significantly higher in patients compared to the control group (Table 3). There was a weak positive correlation between MDA levels and a period of trying for pregnancy (*r* = 0.421, *p* < 0.001) (Table 4).

The total antioxidant potential of the semen expressed as TRAP and the concentration of GSH in infertile men were significantly lower than in fertile men (Table 3). The differences in the studied oxidative stress parameters among people with oligozoospermia, teratozoospermia, and asthenozoospermia were not statistically significant; therefore, a further assessment was performed between the groups of infertile and fertile men. Both TRAP and GSH levels in seminal plasma correlate negatively with the period of trying to have a child (Table 4). Significant positive correlations were observed between TRAP and GSH and seminogram parameters, e.g., concentration, total sperm cell count, and plasma membrane integrity (Table 5 and Table 6).

**Figure 2:**
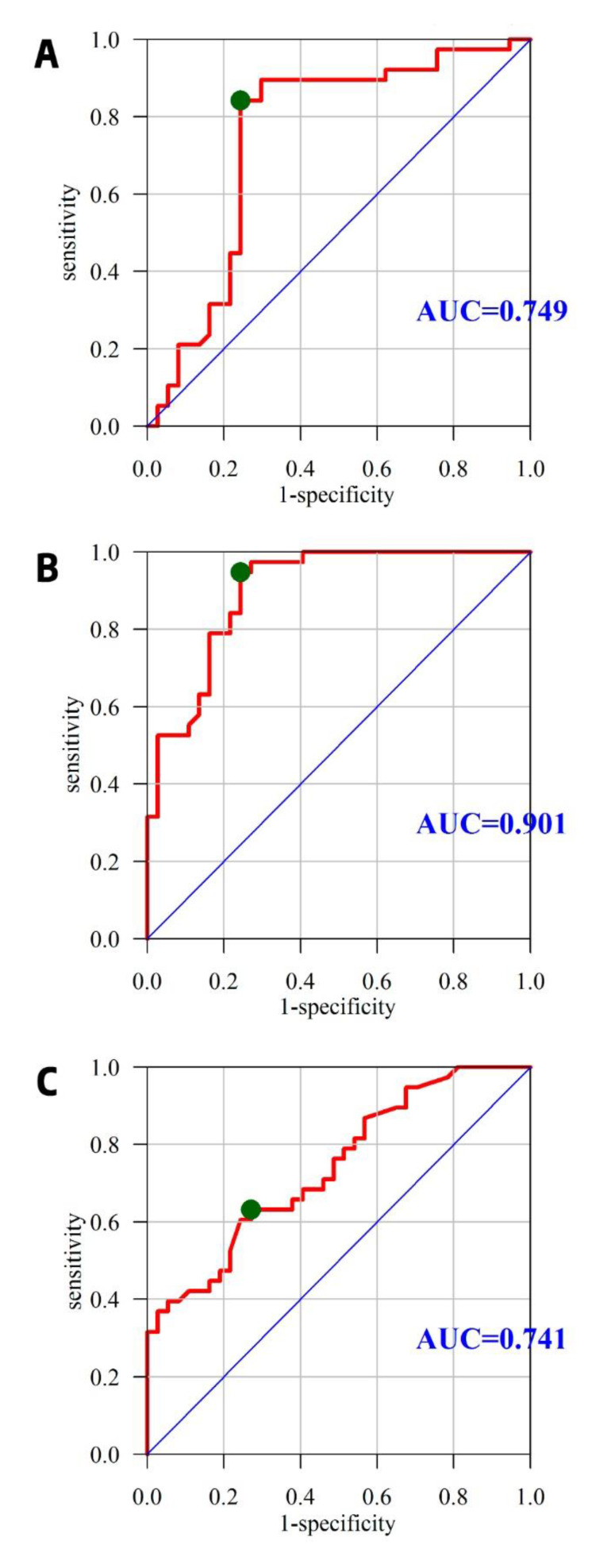
Determination of male infertility predictors from OxS indices using area under the curve (AUC). The cut-off point for tests based on quantitative variables was determined on the basis of the receiver operating characteristic (ROC) curve, choosing the point of the highest sensitivity and specificity. AUC was determined for MDA, TRAP, and GSH. **A**: TRAP (mmol/L); **B**: GSH (μmol/L); **C**: MDA (μmol/mL).

The usefulness of MDA, TRAP, and GSH as predictors of male infertility was evaluated using AUC. The cut-off point for tests based on quantitative variables was determined on the basis of the ROC curve, choosing the point closest to the upper left corner of the graph. The best predictor of fertility problems among the analyzed OxS indices is GSH with an AUC of 0.903, while TRAP and MDA have a lesser predictive potential (Figure 2).

Using the formula proposed by Parikh et al. for TRAP (mM) and GSH (μM), we obtained a total sensitivity for two tests equal to 79.8% and specificity for two tests equal to 94.1% [43]. In comparison, total sensitivity and specificity for three parameters (TRAP, GSH, and MDA) was equal to 50.4% and 98.4%, respectively. The average specificity of the combination of the parameters was higher than the specificity of each of them separately.

## 4. Discussion

Decrease in antioxidant levels and an increase in oxidative damage could induce OxS and causing sperm abnormalities impairing their potential for reproduction is considered one of the major sperm-damaging factors [12,15,17,44]. The mechanisms of this phenomena, however, are not sufficiently explained, while, on the other hand, there is a lack of biomarkers of satisfactory sensitivity and specificity. Meta-analyses of the oxidative stress parameters in infertile men confirm that this topic has been extensively analyzed so far, but the innovation of this research lies the definition of cut-off values for TRAP and GSH as a sum, as well as the relationships between these parameters and changes in the cell membrane. 

Determination of the diagnostic usefulness of the selected OxS indices in the seminal plasma of infertile men and their correlation with seminogram parameters and clinical data seems to be a good strategy for monitoring oxidative stress, which can additionally bring information regarding reproductive potential, especially during the period of trying to initiate pregnancy.

In this study, we explain the usefulness of selected oxidative-antioxidant parameters, i.e., MDA, GSH, and TRAP, as well as their relationship with lifestyle, clinical data, and male infertility. Taking into account a number of published studies on the role of oxidative stress in male infertility, we tried to present a new point of view consisting of a comprehensive approach to the problem of infertility related to lifestyle, eating habits, physical activity, or mental health as factors affecting male reproductive abilities. Therefore, well-defined oxidation and antioxidant potential parameters are presented in the new perspective of the dual role of GSH and TRAP (defense against the negative effects of free radicals, regulation of cell signaling pathways, and reproductive potential), together with an interpretation of their values in seminal plasma. In addition, we present possible interpretations of existing correlations between the selected parameters and male fertility. Due to the significant impact of GSH, TRAP, and MDA on both physiology and pathology of humans and their close relationship with male reproductive function, future research should focus the attention of scientific and clinical communities on the special role of interpreting these biomarkers in monitoring male fertility, which may constitute a new tool of diagnosis and treatment of male infertility.

Seminal plasma treated as a central source of antioxidants protects the sperm against free radical damage. The sperm antioxidant system includes both enzymatic and non-enzymatic components, mutually interacting to provide optimal protective effect against the negative effects of ROS on sperm cells, which are especially sensitive to them due to the high content of unsaturated fatty acids susceptible to lipid peroxidation processes in their cell membrane [45]. This leads to the loss of cellular membrane integrity, its increased permeability, and DNA damage, which accelerates cell apoptosis processes.

It is considered, that the main cellular antioxidant is GSH, a cofactor essential for the operation of basic antioxidant enzymes of sperm, such as GPx, SOD, or CAT, directly binding to ROS through the free sulfhydryl groups present on its surface (-SH). GSH, due to its detoxifying effect on the cytotoxic aldehydes formed in the process of lipid peroxidation, exhibits a protective impact on the sperm cell membrane [46]. Significantly higher levels of GSH were observed in our study in men with normozoospermia compared to men with infertility, suggesting a significant relationship between GSH in the seminal plasma and the integrity of the cell membrane, viability, or sperm morphology, while other studies also report a positive correlation with sperm motility [47]. Our results are consistent with those of Bhardwaj et al., who observed a decrease in GSH in the plasma of patients with oligozoospermia and men with azoospermia compared to normozoospermic individuals [48]. The vast majority of available studies show coherent results, even though there are contradictory ones demonstrating higher concentrations of GSH in patients with azoospermia compared to oligozoospermia or normozoospermia [49]. Several studies demonstrated that the addition of GSH to cryopreserved ejaculate of animals protects the sperm from the harmful effects of ROS accumulated during this process, at the same time improving sperm motility, viability, the integrity of the cell membrane, and sperm DNA, as well as the activity of antioxidant enzymes [50,51,52]. The addition of cysteine to rabbit ejaculate increased biosynthesis of intracellular glutathione and significantly affected cell membrane integrity, acrosomal inviolability or sperm motility [53]. The result of GSH meta-analysis is statistically significant (*p* < 0.001), so evidence was found for differences between the groups. Mean difference (MD) was equal to −13.39, which means that, on average, patients with the problem of infertility had a 13.39 μmol/L lower GSH than patients from the control group. The heterogeneity of studies was significant (*p* < 0.001), so the above results were obtained from a random model. The heterogeneity I² factor was equal to 97.66% [46,47,54,55,56,57,58]. Several studies demonstrated ca. 2-fold decreased GSH concentration in asthenoteratozoospermic patients compared to healthy males [46,47], whereas some did not show any differences in the level of the examined parameter between fertile men and those with idiopathic infertility [54].

MDA levels obtained in our study showed a significant difference between the groups and a correlation with the period of trying for a child. There are several studies reporting the influence of MDA on sperm parameters, even though the method of MDA determination and age of the groups were different [59,60,61]. An analysis of 17 studies on the value of MDA in groups of fertile and infertile men was performed. Its result is statistically significant (*p* < 0.001), with MD values of 1.42 µmol/L and heterogeneity coefficient of 99.75% [57,62,63,64,65,66,67,68,69,70,71,72,73,74,75]. MDA values have been shown to be significantly higher in men with varicocele or infective diseases in the urogenital tract, which correlates with decreased sperm motility [76] and higher percentages of necrotic and apoptotic sperm, and heat stress [77]. This phenomenon can be explained by reduced heat shock proteins and B-cell lymphoma 2 (BCL2) expression, increased BCL2-associated X protein (BAX) expression and higher polymorphism of genes encoding S-glutathione transferase or nitric oxide synthase. 

Several studies have shown beneficial effects of vitamins in men with infertility. The antioxidant effect of vitamin E is primarily associated with the prevention of lipid and DNA peroxidation initiated by ROS and is possible due to the presence of a hydroxyl moiety attached to the chroman ring. The origin of free radicals including lipid peroxidation products, such as MDA, are both mitochondria, as well as non-mitochondrial sources, e.g., NADPH (reduced nicotinamide adenine dinucleotide phosphate) oxidases of cell membranes, such as NADPH oxidase 5 (NOX5), present in mammalian spermatozoa [78]. The intake of vitamin E may improve sperm motility and contribute to the inhibition of lipid peroxidation (lower MDA level) in males with asthenozoospermia after 6 months of therapy [79]. Similarly, combination therapy with vitamin C or zinc also lowers MDA levels [80,81], as well as reduces the percentage of spermatozoa with DNA fragmentation (expressed by DFI, DNA fragmentation index) [82]. The effect of vitamin E on MDA level, sperm motility, and vitality can be enhanced during simultaneous supplementation with selenium [83], which is an integral component of, inter alia, thioredoxin reductase or GPx, which play an important role in antioxidative activity [84]. In addition, it is part of the mitochondrial cover of spermatozoon, which plays an important role in maintaining the proper function and motility of sperm and in the spermatogenesis process [85]. The decrease of concentration of MDA, together with an improvement of sperm parameters, such as motility, mobility, sperm hyperactivation index, and acrosomal response, is observed after the addition of SOD alone or with GSH [86,87]. Such supplementation also correlates with higher TRAP and GSH levels, as well as the reduction in the percentage of dead sperms, and reduces defects to the acrosome [88]. 

As indicated above, supplementation with antioxidants, especially when it is intended for in vitro fertilization, may improve sperm parameters and their antioxidative potential, which can be expressed as TRAP. Infertile men included in this study had lower levels of TRAP and GSH compared to fertile men, which showed positive correlations with seminogram parameters. These results are supported by other literature data, confirming the decrease of GSH level and GPx activity in infertile men [89] or men with low sperm quality [47]. The results of the value of the total antioxidant potential in male semen obtained in this study are consistent with the results of other published papers [14,59,62,63,90,91,92,93], indicating impairment of defense against free radicals in men struggling with the problem of fertility and thus the conception of a descendant [62,63,90,94]. Importantly, this indicator was positively correlated with the parameters of the semen function, such as sperm motility and concentration or sperm with normal morphology [14,63,90,94]. One study reported lack of statistically significant differences between an infertile and fertile group, however, maintaining correlation with seminogram parameters [91]. Another one showed contrary results (higher TRAP values in azoospermic group); however, they can be explained by a variable decrease in the expression of enzymatic antioxidant mechanisms in response to disturbed spermatogenesis in several different male tissues of the reproductive system where the antioxidants production takes place [92].

The oxidative-antioxidant balance parameters we examined were characterized by high predictive power, with AUC values of 0.749 for TRAP (cut-off value 5.523 mmol/L) and 0.901 for GSH (cut-off value 84.124 μmol/L), which is the added value of the presented results, since very few studies have so far paid any attention to this fact [61]. Our study is the first to observe a correlation between TRAP and GSH and the integrity of the sperm cell membrane. Combining the sensitivity and specificity for the two tests, we obtained a better picture of male fertility prediction, related to the imbalance between oxidants and antioxidants in the seminal plasma of fertile and infertile men. This approach assumes that both TRAP and GSH are independent of each other, which additionally supports the presented rule and may help to better predict the disturbance of the oxidative-antioxidant balance of couples using the services of infertility treatment clinics in the future.

Various papers on the ability of plasma semen antioxidants in samples with changed basic semen parameters can be found in the literature. However, the presented results remain incomplete and quite difficult to compare, mainly due to other inclusion and exclusion criteria used in the selection of study groups, small sample size, methodological differences, anatomical anomalies, and differences in age, lifestyle, nutrition patterns, or ethnicity [95,96,97]. The studies on male infertility available in the literature are mainly related to the data from the seminogram, while the innovation of these studies is based on the categorization of patients due to clinical data, excluding patients with clearly defined exclusion criteria. The results on oxidative-antioxidant parameters in fertile and infertile men confirm previous studies. The actual breakthrough and novelty of the conducted research is the definition of cut-off values for TRAP and GSH together, as well as their relationship with the changes in the cell membrane, which have not been studied so far.

The correct level of antioxidants is important in maintaining the basic functions of sperm, while the lack of balance between ROS and antioxidants in the male seminal plasma has a large impact on its fertilizing potential. The multi-source origin of ROS indicates the need for a comprehensive approach to the treatment, in order to reduce complications associated with reproduction which can be induced by OxS. Validation and more frequent use of methods for determining free radical damage in semen and their reference to other parameters constituting the gold standard in male infertility would be a good direction in monitoring male reproductive potential.

This study has several limitations. Logistic regression analysis was not performed. The limitation of this research is a small number of groups that allows the examination of oxidative stress indicators in the multidimensional regression model, which can be used as an additional tool to support clinical decision in predicting infertility. Few men declared the will to participate in the study due to the morning hours of material collection. As another limitation of this study, it should be emphasized that information on the amount of seminal leukocytes, which are the main source of ROS in the ejaculate, was not included. However, we based it on the observations of other researchers who showed that leukocytospermia does not necessarily reflect oxidative damage to sperm DNA [98,99]. Although the percentage of patients with infertility was analyzed within the range previously established in the pilot studies, the number of patients with individual sperm abnormalities was low, which should be considered the main limitation of the study. Greater prospective studies are needed to confirm our preliminary results among men with various sperm abnormalities.

In summary, our study showed for the first time that the combined assessment of TRAP and GSH in the seminal plasma, along with the assessment of other parameters, is a better indicator of male reproductive function than their single analysis. Decreased combined TRAP and GSH is an early predictor of impaired sperm function complicating male infertility, but its diagnostic accuracy appears to be better when compared to seminal TRAP, GSH, or MDA alone. The severity of inflammation (induced by i.a. COVID-19) may significantly affect male reproductive potential not only in the early stages of infection, but it can also shed some light on the persistent effects.

## 5. Conclusions

Our study showed statistically significant differences in the levels of GSH, TRAP, and MDA in the semen of patients with infertility compared to fertile men. These markers, especially in combination increasing their sensitivity and specificity, may have a potential as predictors of male reproductive potential in addition to seminogram performed in routine semen analysis.

We hope that this study will be a premise for further work on the cause-effect role of oxidative stress that is secondary to other changes that may result in male infertility. We think that only such an approach can enable reliable assessment of the scale of the problem related to male infertility.

Further studies on the mutual relations between the antioxidant activity of the seminal plasma and other semen parameters, as well as their impact on clinical parameters, are the direction of our future research. It shall be focused on the assessment of multimodal biomarkers presented in this study in the context of their relationship with epigenetic factors in fertile and infertile men, as well as on monitoring the effectiveness of targeted therapies used to succeed reproductive ability in infertile couples. 

## Figures and Tables

**Table 1:** The results of demographic analysis of factors, such as age, weight, height, and body mass index (BMI), in fertile and infertile groups. *p* < 0.05 indicates significant differences between the groups.

Parameter	Patients		Control		*p* **
	Mean (SD)	Median (Quartiles)	Mean (SD)	Median (Quartiles)	
Age (years)	34.53 (3.95)	35 (32–37)	37.05 (2.46)	37 (36–39)	0.001 *p*
Age of partner (years)	32.13 (3.04)	32 (30–34)	34.59 (2.55)	35 (34–36)	<0.001 *p*
Height (cm)	179.21 (7.33)	180 (173–183.5)	179.03 (6.47)	180 (174–184)	0.909 *p*
Weight (kg)	85.33 (11.6)	89 (74–92.88)	89.62 (7.89)	90 (86–94)	0.122 NP
BMI (kg/m^2^)	26.5 (2.75)	26.26 (24.25–27.99)	28 (2.46)	27.77 (26.88–29.8)	0.016 *p*

** *p* = normality of distribution, Student’s T-test, NP = lack of normality of distribution, Mann–Whitney test.

**Table 2:** Results of the analysis of basic semen parameters, such as volume, concentration, mobility, and vitality, in fertile and infertile groups. P values lower than 0.05 indicate significant differences between the groups.

Parameter	Patients		Control		*P* **
	Mean (SD)	Median (Quartil–es)	Mean (SD)	Median (Quartiles)	
Concentration (mln/mL)	29.16 (10.57)		35.3 (8.03)		0.006 P
Plasma membrane integrity (%)	53.96 (11.11)			82.4 (68.6–90)	<0.001 NP
Viability with Eosin Staining (%)	58.23 (12.31)			84.6 (70–91)	<0.001 NP
Total sperm cell count (mL)		70.8 (43.81–108.7)	109.72 (56.31)		0.063 NP
Volume (mL)		2.5 (2–3.2)	2.9 (1.02)		0.186 NP
Motile sperm progressive cells (%)		29 (21.25–33)	48.97 (15.39)		<0.001 NP
Normal morphology (%)		52.25 (41.25 - 60)	65.21 (13.27)		<0.001 NP
Nonmoving motility of sperm (%)		64 (59.25–71.5)		43 (31–57)	<0.001 NP
Nonprogressive motility of sperm (%)		5 (4–7.75)		5 (3–12)	0.987 NP

** *P* = normality of distribution, Student’s T-test, NP = lack of normality of distribution, Mann–Whitney test.

**Table 3:** The results of selected oxidative stress (OxS) indices of seminal plasma, such as total redox antioxidant potential (TRAP), malondialdehyde (MDA), and glutathione (GSH), in fertile and infertile groups. *p* < 0.05 indicates significant differences between the groups.

Parameter	Patients		Control		*p* **
	Mean (SD)	Median (Quartiles)	Mean (SD)	Median Quartiles)	
TRAP (mmol/L)	4.13 (1.77)			6.93 (5.52–7.42)	<0.001 NP
GSH (µmol/L)		44.14 (38.78–60.21)	109.31 (39.26)		<0.001 NP
Protein (mg/mL)		23.39 (21.94–25.04)		22.31 (21.35–24.52)	0.277 NP
MDA (µmol/mL)		5.29 (4.63–6.89)		4.57 (4.12–5.16)	<0.001 NP

** *P* = normality of distribution, Student’s T-test, NP = lack of normality of distribution, Mann–Whitney test.

**Table 4:** Correlations between the tested parameters and questionnaire data regarding the period of trying to have a child in the examined couples. The strength of the relationship was interpreted in line with the scheme: |r| ≥ 0.9—very strong relationship, 0.7 ≤ |r| < 0.9—strong relationship, 0.5 ≤ |r| < 0.7—moderately strong relationship, 0.3 ≤ |r| < 0.5—weak relationship, and |r| < 0.3—very weak relationship (negligible). Scheme of interpretation according to Hinkle et al. [42].

Parameter	Correlation with the Period of Trying to Have a Child	
	Correlation Coefficient	*P*
Total sperm cell count (mL)	−0.358	0.002
Sum of progressive and non-progressive cells (%)	−0.677	<0.001
Plasma membrane integrity (%)	−0.597	<0.001
Viability with Eosin Staining (%)	−0.506	<0.001
TRAP (mmol/L)	−0.509	<0.001
Protein (mg/mL)	0.095	0.419
GSH (µmol/L)	−0.578	<0.001
MDA (µmol/mL)	0.421	<0.001

**Table 5:** Correlations between semen TRAP level and parameters related to the seminogram among participants of the study. Spearman’s correlation coefficient was used for the analysis of quantitative variables. The strength of the relationship was interpreted in line with the scheme: |r| ≥ 0.9—very strong relationship. 0.7 ≤ |r| < 0.9—strong relationship, 0.5 ≤ |r| < 0.7—moderately strong relationship, 0.3 ≤ |r| < 0.5—weak relationship, and |r| < 0.3—very weak relationship (negligible). Scheme of interpretation according to Hinkle et al. [42].

Parameter	Correlation with TRAP	
	Correlation Coefficient	*P*
Concentration (mln/mL)	0.508	<0.001
Total sperm cell count (mL)	0.476	<0.001
Motile sperm progressive cells (%)	0.631	<0.001
Non-progressive motility of sperm (%)	0.029	0.804
Sum of progressive and non-progressive cells (%)	0.608	<0.001
Plasma membrane integrity (%)	0.759	<0.001
Viability with Eosin Staining (%)	0.663	<0.001
Normal morphology (%)	0.418	<0.001

**Table 6:** Correlations between semen GSH level and parameters related to the seminogram among participants of the study. Spearman’s correlation coefficient was used for the analysis of quantitative variables. The strength of the relationship was interpreted according to the following scheme: |r| ≥ 0.9—very strong relationship, 0.7 ≤ |r| < 0.9—strong relationship, 0.5 ≤ |r| < 0.7—moderately strong relationship, 0.3 ≤ |r| < 0.5—weak relationship, and |r| < 0.3—very weak relationship (negligible). Scheme of interpretation according to Hinkle et al. [42].

Parameter	Correlation with GSH	
	Correlation Coefficient	*P*
Concentration (mln/mL)	0.452	<0.001
Total sperm cell count (mL)	0.394	<0.001
Motile sperm progressive cells (%)	0.68	<0.001
Non-progressive motility of sperm (%)	0.022	0.849
Sum of progressive and non-progressive cells (%)	0.661	<0.001
Plasma membrane integrity (%)	0.795	<0.001
Viability with Eosin Staining (%)	0.72	<0.001
Normal morphology (%)	0.415	<0.001

## Supplementary Materials

The following are available online at https://www.mdpi.com/2077-0383/9/6/1785/s1, Supplementary Material S1: Questionnaire Data.
