# Supplementary material for: Sperm Antioxidant Biomarkers and Their Correlation with Clinical Condition and Lifestyle with Regard to Male Reproductive Potential
Source: J Clin Med. 2020 Jun 8;9(6):1785. doi: 10.3390/jcm9061785 (PMC7355971; doi:10.3390/jcm9061785)
Supplement: Supplementary file 1 [file jcm-09-01785-s001.pdf]

Journal of Clinical Medicine

Article

Supplementary Material S1: Questionnaire data

Table S1. Study questionnaire

Questionnaire number				
Participant's age		Height		
Partner's age		Weight		
Ethnicity		The cause of infertility		
Working conditions	Sitting	Standing	Manual worker	
Harmful working conditions, if applicable	High temperature	Low temperature	Pesticides, chemicals	Radiation
Other exposure to high temperatures	Sauna	Thermal pools	Excessive sunbathing	Other
Physical activity	None	Once a week	3 times a week	Daily
Diet	No limitations	Protein	Vegetarian	Other
Drugs:				
Alcohol	None	Occasionally	Frequently	Daily
Cigarettes	None	Occasionally	2-5 daily	>6 daily
Narcotics	None	Occasionally	Frequently	Daily
Coffee	None	Occasionally	1-2 cups daily	>3 cups daily
Fruits and vegetables intake	None	Less than once a week	1-2 times a week	Daily

Table S2. History of infertility

Do you have any children?	Yes	No
Number of intercourses per week:		
How long for are you trying to conceive?		
Do you have any troubles with erection?	Yes	No
Is there any blood in semen?	Yes	No
Is ejaculation painful?	Yes	No
Did you experience testicles swelling, enlargement or displacement?	Yes	No
Did you experience any troubles with ejaculation, premature ejaculation or lack of ejaculate?	Yes	No
Does infertility occur in your family (brothers or sisters)?	Yes	No
Has your father been treated from any urological condition?	Yes	No

Table S3. Disease history

Autoimmune diseases	Yes	No
Thyroid disease	Hyperthyroidism<br>Hypothyroidism	No
Diabetes	Type 1<br>Type 2	No
Viral hepatitis	Type A	No

	Type B	
	Type C	
Urological diseases	Nephritis<br>Kidney stone disease (urolithiasis)<br>Difficulty in urinating<br>Prostate diseases<br>Bacterial urinary tract infection	No
Genetic diseases in family	X-linked (linked with sex)<br>Autosomal	No
Rheumatic diseases	Rheumatoid arthritis<br>Systemic lupus erythematosus<br>Sjögren syndrome<br>Ankylosing spondylitis<br>Other	No
Mental disorders	Depression<br>Bipolar affective disorder<br>Schizophrenia<br>Anxiety disorders<br>Chronic stress	No
Cancer	Malignant<br>Benign	No
Hypertension	Yes	No
Sexually transmitted diseases	Syphilis / gonorrhea / chlamydiosis / AIDS / herpes virus / human papillomavirus / scabies / lice / trichomoniasis / candidiasis / non-gonococcal urethritis	No

Table S4. History of treatment

Did you take any antibiotics in the last 6 months?	Yes	No
Do you take any drugs chronically? If so, please enter their names	Yes	No

Study questionnaire – results

Table S5. Working conditions

Parameter (Mean±SD)	Sitting N=48	Standing N=9	Manual worker N=18	p*
Total sperm cell count (mL)	91.44±55.21	114.01±44.24	109.46±79.63	0.329
Sum of progressive and non-progressive cells (%)	43.88±18.04	56.11±22.3	44.72±17.41	0.317
Plasma membrane integrity (%)	63.36±17.61	76.18±20.39	67.18±14.97	0.154
Viability with Eosin Staining (%)	65.6±18.11	77.2±18.28	70.78±13.01	0.093
TRAP (mmol/L)	4.71±2.25	6.17±1.87	5.29±1.8	0.191
Protein (mg/mL)	23.75±5.46	24.81±5.42	24±3.41	0.570

GSH (μmol/L)	73.17±39.43	107.52±52.79	81.2±41.3	0.120
MDA (μmol/mL)	5.39±1.06	4.81±0.8	4.98±1.17	0.123

* lack of normality of distribution, non-parametric analysis, Kruskal-Wallis test

Table S6. High temperature

Parameter (Mean±SD)	Yes N=25	No N=50	p*
Total sperm cell count (mL)	78.7±50.22	108.36±63.6	0.056
Sum of progressive and non-progressive cells (%)	44.16±20.27	46.24±17.87	0.438
Plasma membrane integrity (%)	62.94±17.51	67.26±17.69	0.208
Viability with Eosin Staining (%)	66.08±17.01	69.31±17.5	0.186
TRAP (mmol/L)	4.16±2.14	5.46±2.02	0.013
Protein (mg/mL)	23.28±5.26	24.27±4.87	0.094
GSH (μmol/L)	71.21±42.56	83.23±42.26	0.206
MDA (μmol/mL)	5.46±1.11	5.1±1.05	0.129

* lack of normality of distribution, non-parametric analysis, Kruskal-Wallis test

Table S7. Low temperature

Parameter (Mean±SD)	Yes N=10	No N=65	p*
Total sperm cell count (mL)	89.04±63.86	99.92±60.68	0.533
Sum of progressive and non-progressive cells (%)	38.4±15.76	46.65±18.86	0.190
Plasma membrane integrity (%)	62.16±16.57	66.38±17.84	0.346
Viability with Eosin Staining (%)	64.05±15.29	68.88±17.6	0.268
TRAP (mmol/L)	3.93±2.11	5.2±2.11	0.089
Protein (mg/mL)	23.1±3.37	24.07±5.2	0.876
GSH (μmol/L)	67.45±40.9	81.03±42.71	0.400
MDA (μmol/mL)	5.58±1.22	5.17±1.05	0.268

* lack of normality of distribution, non-parametric analysis, Kruskal-Wallis test

Table S8. Any harmful working condition

Parameter (Mean±SD)	Yes N=31	No N=44	p*
Total sperm cell count (mL)	81.57±55.17	110.38±62.32	0.037

Sum of progressive and non-progressive cells (%)	43.71±19.93	46.84±17.71	0.398
Plasma membrane integrity (%)	63.77±17.27	67.26±17.94	0.256
Viability with Eosin Staining (%)	66.83±16.29	69.23±18.09	0.307
TRAP (mmol/L)	4.28±2.11	5.55±2.02	0.013
Protein (mg/mL)	23.16±4.78	24.48±5.11	0.079
GSH (µmol/L)	72.39±43.36	84.03±41.64	0.188
MDA (µmol/mL)	5.39±1.14	5.1±1.02	0.198

* lack of normality of distribution, non-parametric analysis, Kruskal-Wallis test

Table S9. Physical activity

Parameter (Mean±SD)	Never (A) N=13	1-3 times a week (B) N=48	Every day (C) N=14	p*
Total sperm cell count (mL)	78.68±90.89	100.48±55.59	109.96±41.02	0.045 (C>A)
Sum of progressive and non-progressive cells (%)	38±23.56	46.56±17.52	49.07±16.48	0.155
Plasma membrane integrity (%)	58.38±13.61	66.35±18.19	70.88±17.84	0.148
Viability with Eosin Staining (%)	64.46±14.64	67.94±18.15	72.76±16.65	0.412
TRAP (mmol/L)	3.47±2.01	5.36±2.09	5.31±1.89	0.019 (B>A)
Protein (mg/mL)	22.29±5.39	23.98±4.66	25.34±5.62	0.036 (C>A)
GSH (µmol/L)	66.88±35.16	80.84±42.52	85.11±48.85	0.552
MDA (µmol/mL)	5.73±1.23	5.08±0.93	5.22±1.29	0.267

* lack of normality of distribution, non-parametric analysis, Kruskal-Wallis test

Table S10. Drinking coffee

Parameter (Mean±SD)	Never/ occasionally N=47	Every day N=28	p*
Total sperm cell count (mL)	98.26±49.72	98.83±76.9	0.607
Sum of progressive and non-progressive cells (%)	46.51±14.73	43.93±23.95	0.188
Plasma membrane integrity (%)	66.64±18.36	64.44±16.58	0.573
Viability with Eosin Staining (%)	68.55±18.43	67.71±15.52	0.603

TRAP (mmol/L)	5.54±1.99	4.17±2.14	0.007
Protein (mg/mL)	23.1±2.76	25.34±7.21	0.948
TRAP (mmol/mg of protein)	243.18 ±90.13	169.93 ±99.14	0.003
GSH (µmol/L)	87.32±46.74	65.62±30.23	0.072
MDA (µmol/mL)	5.06±1.09	5.49±1	0.036

* lack of normality of distribution, non-parametric analysis, Kruskal-Wallis test

Table S11. Fruits and vegetables intake

Parameter (Mean±SD)	Less than every day N=33	Every day N=42	p*
Total sperm cell count (mL)	81.3±53.14	111.97±63.57	0.021
Sum of progressive and non-progressive cells (%)	43.55±21.81	47.12±15.72	0.132
Plasma membrane integrity (%)	61.87±17.26	68.92±17.5	0.114
Viability with Eosin Staining (%)	64.24±16.95	71.38±17.11	0.115
TRAP (mmol/L)	4.12±2.09	5.74±1.92	0.001
Protein (mg/mL)	23.37±4.53	24.39±5.33	0.191
GSH (µmol/L)	67.3±38.68	88.58±43.39	0.059
MDA (µmol/mL)	5.57±1.03	4.95±1.04	0.006

* lack of normality of distribution, non-parametric analysis, Kruskal-Wallis test

Table S12. Having offspring

Parameter (Mean±SD)	Yes N=41	No N=34	p*
Total sperm cell count (mL)	100.79±51.23	95.67±71.34	0.44
Sum of progressive and non-progressive cells (%)	54.78±17.56	34.41±12.95	<0.001
Plasma membrane integrity (%)	73±18.17	57.16±12.48	<0.001
Viability with Eosin Staining (%)	73.85±18.96	61.46±12.18	0.002
TRAP (mmol/L)	5.72±2.08	4.19±1.92	0.002
Protein (mg/mL)	23.49±4	24.48±5.99	0.463
GSH (µmol/L)	100.76±44.79	53.25±18.45	<0.001
MDA (µmol/mL)	4.88±0.93	5.63±1.09	0.001

* lack of normality of distribution, non-parametric analysis, Kruskal-Wallis test
